# P-1453. Characterization of longitudinal humoral and cellular immunogenicity following 2 or 3 doses of an mRNA-based cytomegalovirus vaccine in healthy adults

**DOI:** 10.1093/ofid/ofaf695.1639

**Published:** 2026-01-11

**Authors:** Jaap Oostendorp, Kai Wu, Cathal Harmon, Shannon McGrath, Angela Nalwoga, Bethany Girard, Lori Panther, Robert Paris

**Affiliations:** Moderna, Inc, Cambridge, MA; Moderna, Inc., Cambridge, Massachusetts; Moderna, Cambridge, Massachusetts; Moderna, Inc., Cambridge, Massachusetts; Moderna, Cambridge, Massachusetts; Moderna, Inc., Cambridge, Massachusetts; Moderna, Inc., Cambridge, Massachusetts; Moderna, Inc., Cambridge, Massachusetts

## Abstract

**Background:**

Cytomegalovirus (CMV) is a common herpesvirus that poses a substantial unmet medical need for congenital CMV and CMV disease in immunocompromised individuals. A preventive vaccine remains a public health priority. Our previous data have shown that 3 doses of mRNA-1647, an investigational mRNA-based CMV vaccine, induce humoral and cellular immune responses. These results provided support for advancing a 3-dose schedule into a Ph3 efficacy study. Here we compared 2- and 3-dose schedules and assessed longitudinal responses for at least one year after the last vaccination.

**Methods:**

In this clinical study (NCT05397223), we compared the immunogenicity of a 2-dose (0, 2 months) and 3-dose (0, 2, 6 months) regimen of mRNA-1647, encoding the CMV glycoprotein B and pentameric complex, in healthy individuals 18-49 years of age. We evaluated humoral immune response in CMV-seropositive and -seronegative participants up to 12 months after the last vaccination by assessing neutralizing Ab (nAb) titers against fibroblast and epithelial infection. In addition, cellular responses were evaluated by intracellular cytokine staining to characterize the magnitude of the antigen-specific T cell response to gB, gH, and gL-UL peptide pools.

**Results:**

Both the 2-dose and 3-dose regimens of mRNA-1647 elicited robust and durable nAb titers, with higher fold-rises from baseline in CMV-seronegative than in CMV-seropositive individuals up to 12 months after the last vaccination. The magnitude of the nAb response at 12 months was higher following 3 doses of mRNA-1647 compared to 2 doses, especially in CMV-seronegative participants. Similarly, higher frequencies of polyfunctional antigen-specific T cell responses were observed at 12 months after 3 doses of mRNA-1647 when compared to 2 doses.

**Conclusion:**

Our results show that mRNA-1647 elicits strong humoral and cellular immune responses with persistence demonstrated up to 12 months after last vaccination. Higher responses seen at peak and later timepoints support selection of the 3-dose regimen in the ongoing Phase 3 efficacy trial. Assessment of a 0-6 month schedule is ongoing.

**Disclosures:**

Jaap Oostendorp, PhD, Moderna, Inc.: Employee|Moderna, Inc.: Stocks/Bonds (Public Company) Kai Wu, PhD, Moderna, Inc: Employee|Moderna, Inc: Stocks/Bonds (Public Company) Cathal Harmon, PhD, Moderna, Inc: Employee|Moderna, Inc: Stocks/Bonds (Public Company) Shannon McGrath, MS, Moderna, Inc.: Employee|Moderna, Inc.: Stocks/Bonds (Public Company) Angela Nalwoga, MS, Moderna, Inc: Employee|Moderna, Inc: Stocks/Bonds (Public Company) Bethany Girard, Ph.D., Moderna, Inc.: Employee|Moderna, Inc.: Stocks/Bonds (Public Company) Lori Panther, MD, MPH, Moderna, Inc: Employee|Moderna, Inc: Stocks/Bonds (Public Company) Robert Paris, MD, Moderna, Inc.: Employee|Moderna, Inc.: Stocks/Bonds (Public Company)

